# Candidate Genes for Proliferative Diabetic Retinopathy

**DOI:** 10.1155/2013/540416

**Published:** 2013-08-27

**Authors:** Daniel Petrovič

**Affiliations:** ^1^Institute of Histology and Embryology, Medical Faculty, University Ljubljana, Korytkova 2, 1105 Ljubljana, Slovenia; ^2^Zavod Srce, Dunajska 106, 1000 Ljubljana, Slovenia

## Abstract

Several candidate genes have been so far implicated in the pathogenesis of proliferative diabetic retinopathy (PDR) in subjects with type 2 diabetes. Since the principal pathogenetic mechanisms for diabetic retinopathy (DR) and PDR are different, the main pathogenetic mechanism in DR is increased vascular permeability, whereas in PDR the crucial pathogenetic mechanisms are fibrosis and neoangiogenesis. Due to that fact, different candidate genes are expected to be involved in the development of either DR or PDR. None of the candidate genes, however, can be fully and solely responsible for the development of PDR and for DR progression into PDR. Epigenetic mechanisms are expected to be involved in the pathogenesis of PDR as well. Gene polymorphisms responsible for PDR and epigenetic mechanisms responsible for PDR are reviewed in this paper.

## 1. Introduction

Changes in dietary habits and lifestyles associated with rapid economic growth have dramatically increased the incidence of diabetes, obesity, and related vascular complications [1, 2]. Both type 1 and type 2 diabetes are associated with hyperglycaemia, oxidant stress, and inflammation and significantly increased risk for macrovascular complications and microvascular complications [1, 2].

Diabetic retinopathy (DR) is associated with both environmental and genetic factors. Several metabolic abnormalities are implicated in its pathogenesis; however, the exact mechanism remains to be determined. Several studies have been devoted to the evaluation of environmental and genetic factors related to DR and proliferative DR (PDR), whereas much less is known about epigenetic mechanisms [3–7]. 

Environmental risk factors for DR and PDR are well known, including duration of diabetes, glycaemic control, hypertension, and other environmental factors, whereas the genetic risk factors for the development and progression of DR into PDR are only beginning to be understood [8–11] (Table 3). There is growing evidence, however, that PDR has a genetic predisposition [12–19]. 

The progression of diabetic retinopathy DR to PDR is a serious complication of diabetes [20]. Ischemia-induced retinal neovascularization in association with the outgrowth of fibrovascular epiretinal membranes at the vitreoretinal interface is a hallmark feature of PDR and often leads to severe visual loss due to haemorrhage and/or tractional retinal detachment [21]. In PDR, extensive proliferation of new vessels (neoangiogenesis) often leads to vitreous haemorrhage, retinal detachment, and neovascular glaucoma. A surgical procedure called vitrectomy can alleviate to a limited extent some of the complications of PDR, thereby preventing visual loss.

## 2. Pathogenesis of DR and PDR

Diabetic retinopathy is characterized by increased vascular permeability, haemostatic abnormalities, endothelial dysfunction, increased tissue ischemia, and neoangiogenesis [2]. The pathogenetic mechanisms of DR are very complex. Many hyperglycemia-induced metabolic abnormalities are implicated in its pathogenesis, such as alteration in retinal blood flow, hemostatic abnormalities, metabolic changes, increased oxidative stress, increased polyol pathway flux, activation of protein kinase C isoforms, increased hexosamine pathway flux, and increased advanced glycation endproduct formation, nonenzymatic glycosylation of collagen, and other tissue proteins, which are observed during long-term hyperglycemia [2, 9]. Moreover, many systemic abnormalities in diabetes affect the platelet function and thrombotic system, and these may be involved in the development of DR and/or PDR. These changes are hyperreactive platelets, decreased vascular prostacyclin production, endothelial dysfunction resulting in increased circulating levels of von Willebrand factor and leukocyte adhesion molecules, hypercoagulability, and decreased fibrinolysis. Increased levels of intercellular adhesion molecule-1 and of plasminogen activator inhibitor-1 mRNA as well as decreased levels of tissue plasminogen activator are specifically found in retinal vessels of diabetic compared with nondiabetic individuals [22, 23].

Inflammation, fibrosis, and angiogenesis are processes involved in the pathogenesis of PDR [24]. The causal relationship between inflammation and angiogenesis in PDR is now widely accepted [25]. The emerging focus in PDR research is on the link between chronic, low-grade inflammation and angiogenesis. Although the exact mechanism of the development of retinopathy remains elusive, growth factors, cytokines, and chemokines were implicated in the progression of DR and in the development of angiogenesis [26, 27]. In normal ocular tissue, angiogenic hemostasis is controlled by the balance between stimulators of angiogenesis, such as VEGF, and inhibitors of angiogenesis, such as pigment epithelium-derived factor, whereas in PDR this balance is shifted toward increased angiogenesis [28]. Retinal hypoxia induces increased expression of several genes, such as VEGF, erythropoietin (EPO), and cytokines, leading to fibrosis and angiogenesis [26]. Recently, increased expression of VEGF and EPO and decreased expression of pigment epithelium-derived factor have been demonstrated in vitreous fluid in patients with PDR in comparison to control group [27]. Moreover, vitreous levels of EPO and VEGF correlated with the HbA1c in patients with PDR [27] implicating the effect of diabetes control on PDR progression.

Higher levels of high-mobility group box-1 protein (HMGB1), vascular endothelial growth factor (VEGF), interleukin 8, intercellular adhesion molecule (ICAM-1), vascular cell adhesion molecule (VCAM-1), soluble vascular endothelial-cadherin, vascular adhesion protein, erythropoietin (EPO), soluble endoglin, and chemokines MCP-1 and IP-10 have been recently reported in vitreous fluid from PDR patients compared to nondiabetic patients [16, 27, 29–32]. In vascular endothelial cells and in the retina, expression of EPO, a glycoprotein that stimulates erythropoiesis and angiogenesis, and expression of EPO receptors were demonstrated [33, 34]. Moreover, McVicar and coworkers [35] have recently demonstrated that treatment with an EPO-derived peptide in fully established diabetes could significantly protect against neuroglia and vascular degenerative pathology without altering hematocrit or exacerbating neovascularization (2011).

It is speculated that HMGB1 might be an important link between chronic low-grade inflammation and angiogenesis. HMGB1 functions as a proinflammatory cytokine and exhibits angiogenic effects [25, 36]. The binding of HMGB1 on the receptor for advanced glycation endproducts (RAGE) activates the transcription factor nuclear factor kappa B (NF-*κ*B). Finally, the activation of NF-*κ*B induces the expression of various leukocyte adhesion molecules and proinflammatory cytokines, chemokines, and angiogenic factors [25]. 

Additionally, endoglin (sEng) has recently been reported to regulate angiogenesis beside affecting endothelial cell function [27, 37]. In vitro and in vivo studies demonstrated that sEng is capable of inhibiting angiogenesis [37]. Abu El-Asrar and coworkers have recently demonstrated a significant negative correlation between sEng levels and the levels of sVE- cadherin in the vitreous from patients with PDR [27]. These findings suggest a lower angiogenic activity in patients with higher levels of sEng and that the upregulation of sEng in the vitreous fluid from patients with PDR may be a protective antiangiogenesis eye response to suppress the progression of PDR [27].

Moreover, oxidative stress has also been recently reported to affect the development of PDR [32]. Vascular adhesion protein (VAP)-1 has been implicated as a possible link between inflammation and oxidative stress in PDR [32]. Increased vitreous levels of VAP-1 have been demonstrated in patients with PDR ([32]. Moreover, in vitro studies have demonstrated increased sVAP-1 after stimulation with high glucose or inflammatory cytokines, such as TNF-*α* and IL-1*β* [32]. Furthermore, matrix metalloproteinase-2 and -9, type IV collagenases, were the key molecules to mediate the protein cleavage of VAP-1 from retinal capillary endothelial cells. Murata and coworkers were the first to provide evidence on the link between sVAP-1 and type IV collagenases in the pathogenesis of PDR [32].

Recently, the significant decrease of angiogenic factors (angiopietin-2, HGF, VEGF, and EPO) in the vitreous fluid after vitrectomy has been reported suggesting that vitrectomy shifts the eye towards an antiangiogenic environment [38, 39].

## 3. Candidate Genes for PDR

Several candidate genes have been so far implicated in the pathogenesis of PDR in subjects with type 2 diabetes (Table 1) [12–19]. Since the principal pathogenetic mechanisms for DR and PDR are different, namely, increased vascular permeability in DR and primarily fibrosis and neoangiogenesis in PDR, different candidate genes are expected to be involved in the development of PDR. Moreover, several gene polymorphisms have been so far reported to be associated with PDR and to be potential genetic markers for PDR in subjects with type 2 diabetes [12–19].

## 4. Gene Polymorphisms of Growth Factors

The substantial overexpression of VEGF was demonstrated in fibrovascular membranes in patients with proliferative diabetic retinopathy, suggesting that this molecule might contribute to the development of PDR [40]. Expression of growth factors, such as VEGF, basic fibroblast growth factor (BFGF), and insulin-like growth factor (IGF), is influenced by single-nucleotide polymorphisms (SNPs) in these genes [41–52]. Several studies have so far demonstrated the importance of gene polymorphisms VEGF, BFGF, and IGF in the pathogenesis of PDR (Table 2) [41–52]. So far, 634C/G polymorphism of the VEGF was the most often tested polymorphism; however, in all studies 634C/G polymorphism failed to be associated with PDR, whereas other polymorphisms have been tested more rarely; therefore, confirmatory studies are warranted.

## 5. Gene Polymorphisms of Oxidative Stress Genes and PDR

Several studies have so far reported an association between risk genotypes of the oxidative stress genes and PDR in type 2 diabetes (Table 3) [19, 53–55]. In two large studies, an association has recently been reported, namely, between either aa genotype of the eNOS 4a/b polymorphism or haplotype A val Ins and PDR in Caucasians [19, 53]. So far, no polymorphism of the genes affecting oxidative stress has been confirmed in another population in patients with PDR.

## 6. Genes Affecting the Iron Metabolism

Genes involved in iron metabolism (hemochromatosis gene, EPO gene) have also been implicated in the pathogenesis of PDR (Table 4) [35, 56–58]. The first report about the role of iron metabolism in the development of PDR was made in 2003. The authors reported the association of the C282Y in the hemochromatosis (HFE) gene with proliferative diabetic retinopathy in Caucasians with type 2 diabetes [57]; however, the authors did not propose the mechanism behind this finding. It is speculated that changes in iron metabolism were involved in the PDR development via oxidative stress. Moreover, it was reported that expression of EPO was influenced by single-nucleotide polymorphisms (SNPs) in the EPO gene [56]. So far, however, different polymorphisms of the genes affecting iron metabolism have been tested on various populations in patients with PDR, and confirmatory studies are warranted.

## 7. Gene Polymorphisms of Cytokine Genes and PDR

Overexpression of cytokines has recently been reported in in vitreous fluid in patients with PDR, and cytokines are implicated in the pathogenesis of fibrosis and angiogenesis [24, 26]. Moreover, several studies have so far reported an association between risk genotypes of the cytokine genes and PDR in type 2 diabetes (Table 5) [48–50, 59–63]. So far, however, none of the risk genotypes has been reported to be associated with PDR in more than one population, suggesting that studies of the same polymorphisms on different populations are needed.

## 8. Gene Polymorphisms of the Adhesion Molecules and PDR

The substantial overexpression of adhesion molecules ICAM-1, VCAM-1, and of VEGF was demonstrated in fibrovascular membranes in patients with proliferative diabetic retinopathy, suggesting that these molecules might contribute to the development of PDR [40]. Few studies have so far tested possible association between risk genotypes of adhesion molecules and PDR in type 2 diabetes (Table 6) [18, 64]. One risk genotype and one risk allele were reported to be associated with the development PDR, EE genotype of the IKAM K469E polymorphism in Caucasians, and *K* allele of the IKAM K469E in Chinese population (Table 6) [18, 64], indicating potential interracial differences.

## 9. Gene Polymorphisms of the Polyol Pathway and PDR

Few studies have so far tested the possible association between risk genotypes of the polyol and PDR in type 2 diabetes (Table 7) [65–67]. Two risk genotypes were reported to be associated with the development of PDR, namely, z-4 genotype of the (AC⁡)_*n*_ polymorphism and CC genotype of the −106C>T polymorphism of the aldose reductase (Table 7) [65, 66].

## 10. Gene Polymorphisms of the Renin-Angiotensin System and PDR

Few studies have so far tested the possible association between risk genotypes of the renin-angiotensin system and PDR in type 2 diabetes; however, they failed to demonstrate an association (Table 8) [68–71]. According to these results, it may be concluded that RAS system does not have an important part in the pathogenesis of PDR.

## 11. Proteomics 

Beside genomics, other omics technologies have offered important information trying to reveal the pathogenetic mechanisms in PDR. So far, especially proteomics has been very helpful in revealing several biological pathways and defining potential new drug targets [72–74].

Biological pathway analysis of the study reported in 2008 revealed that the vitreous contains 30 proteins associated with the kallikrein-kinin system, coagulation, and complement systems. Seven of them (complement C3, complement factor I, prothrombin, alpha-1-antitrypsin, antithrombin III, angiotensinogen, and peroxiredoxin-1) were increased in PDR vitreous compared with control vitreous, whereas decreased levels of extracellular superoxide dismutase and neuroserpin were demonstrated in PDR vitreous [75]. Recently, Wang and coworkers [73] have revealed 44 proteins involved in 56 biological pathways in PDR. The most remarkable pathways differentially represented between PDR and normal vitreous were the glycolysis/gluconeogenesis, complement and coagulation cascades, gap junction, and phagosome pathways. The differential expressions of angiopoietin-related protein 6, apolipoprotein A-I, estrogen receptor alpha, and tubulin were confirmed by Western blot analysis [75]. Since improved and more sensitive techniques for the proteome analysis are emerging, new data are expected [76].

## 12. Epigenetic factors and DR

So far, several candidate genes have been implicated in the pathogenesis of diabetic retinopathy via complex interactions of environmental and genetic factors. None of them, however, can be fully and solely responsible for the development of diabetic retinopathy and for DR progression into PDR.

Good glycemic control, if started in the initial stage of diabetes, prevents the development of retinopathy, but if reinstituted after a period of poor control, fails to halt its development, suggesting a metabolic memory phenomenon [4]. Patients in the conventional treatment regimen during the diabetes complications and control trial had a higher incidence of complications several years after switching to intensive therapy than the patients in intensive control [77, 78]. Studies in rats have demonstrated that the retina continues to experience oxidative stress, MnSOD remains compromised, and NF-*κ*B is activated for at least 6 months after reinstitution of good glycemic control that has followed 6 months of poor control. This phenomenon has recently been reported to be due to the global acetylation of retinal histone H3 [4]. The epigenetic regulation has been demonstrated in manganese superoxide dismutase gene [4], whereas it has not been studied in any integrin gene yet. A similar mechanism (i.e., global acetylation of retinal histone H3) has also been proposed in the pathogenesis of the progression of DR.

Epigenetic changes occur without alterations in the DNA sequence and can affect gene transcription in response to environmental changes and nutrition. Transition from the active to the inactive state of chromatin is the central mechanism of gene regulation, and this is defined as epigenetic factor. Several pathways may be involved in the epigenetic regulation, that is, DNA methylation, histone acetylation, and noncoding RNAs or microRNAs [3, 4]. 

Modulation of epigenetic changes by pharmaceutical means may provide a potential strategy to retard the progression of DR. Beside intense medical management, these strategies include dietary measures and the introduction of epigenetic drugs, such as inhibitors of DNA methylation and histone demethylases.

## 13. Conclusions

Changes in dietary habits and lifestyles associated with rapid economic growth have dramatically increased the incidence of diabetes and related macrovascular and microvascular complications. Several factors, such as hyperglycaemia, oxidative stress, inflammation, and platelet dysfunction, are implicated in the pathogenesis of diabetic retinopathy. So far, several studies have demonstrated the importance of several environmental and genetic factors. However, larger cross-sectional studies and well-powered meta-analyses are needed to identify more successfully underlying genetic variants for PDR. However, much less is known about gene-environment interactions and epigenetic changes.

Alarming estimates indicate that the rate of diabetes and associated complications (including DR) are rapidly increasing; therefore, additional strategies to arrest these trends are needed. Beside intense medical management, these strategies include dietary measures and the introduction of epigenetic drugs, such as inhibitors of DNA methylation and histone demethylases.

Finally, based on current knowledge, optimising the medical management of diabetic retinopathy should address the control of glycaemia, blood pressure, and lipids, and specific therapies using fenofibrate with a statin and candesartan should be considered.

It is generally accepted that susceptibility to DR and PDR is most likely determined by a large number of relatively common allelic variants, possibly interlinked and interacting with environmental influences, each individually conferring a modest increase in relative risk. Identifying these variants in an individual and utilizing the appropriate medical management seem promising in the prevention and treatment of DR and other diabetic vascular complications, taking one step further towards personalized medicine.

## Figures and Tables

**Table 1:** Pathways and genes implicated in the pathogenesis of PDR.

Pathway/systems	Gene
Growth factors	Vascular endothelial growth factor
	Basic fibroblast growth factor
	Insulin-like growth factor

Oxidative system	Manganese superoxide dismutase
	Catalase myeloperoxidase
	Glutathione S-transferase
	NADPH oxidase
	Endothelial nitric oxide synthase
	Inducible nitric oxide synthase

Inflammatory genes	Interleukins
	Tumor necrosis factor

Adhesion molecules	Intercellular adhesion molecule
	Vascular cell adhesion protein
	Platelet endothelial cell adhesion molecule

Polyol pathway	Aldose reductase/sorbitol dehydrogenase

Renin-angiotensin system	Renin
	Angiotensinogen
	Angiotensin 1 converting enzyme
	Aldosterone

Advanced glycation end products	Receptor for advanced glycation end products

Peroxisome proliferator-activated receptor	Peroxisome proliferator-activated receptor
	Coactivator for peroxisome proliferator-activated receptor

Thrombotic system	Fibrinogen

Platelet function	Integrin

Extracellular matrix homeostasis	Matrix homeostasis genes
	Matrix metalloproteinase

Hormones/vitamins	Growth hormone
	Vitamin D

Undefined	Glucose transporter-1
	Growth hormone

**Table 2:** The reported studies of growth factor genes and proliferative diabetic retinopathy in subjects with type 2 diabetes.

Population	Subjects with type 2 diabetes (*n*)	Polymorphism (rs)	Risk genotype/allele	OR (*P* value)^1^	Reference
Japanese population	536 (PDR versus NDR dm without DR)	VEGF^2^ −634C/G	CC—no association	1.5 (0.08)	[46]
Slovak population (Caucasians)	245 (PDR versus dm without PDR)	TGF-beta^3^ +915G/C (R25P)	RR genotype	2.89 (<0.01)	[42]
English population (Caucasians)	267 (PDR versus non-PDR)	VEGF^2^ −460C/T, VEGF^2^ +405C/G	CC; no association	3.7 (0.02) NA^4^	[43]
Indian population	208 (PDR versus non-PDR)	IGF^5^-(CA)_*n*_	18-(AC) repeats	2.8 (<0.05)	[51]
Slovak population (Caucasians)	488 (PDR versus non-PDR versus dm without DR)	bFGF^6^ −754C/G	CC	NA^4^ (0.006)	[44]
Slovene population (Caucasians)	349 (PDR versus dm without DR)	VEGF^2^ −634C/G	CC—no association	1.1 (0.7)	[16]
Polish population (Caucasians)	215 (PDR versus non-PDR versus dm without DR)	VEGF^2^ −634C/G, VEGF^2^ +460C/T	No association	NA^4^	[52]
Slovene population (Caucasians)	313 (PDR versus dm without DR)	bFGF^6^: −553T/A, −834T/A, −921C/G	−553T/A AT genotype; −834T/A AT genotype	2.0 (0.03); 0.4 (0.01); no association; consecutively	[17]
South Korean population	398 (PDR versus non-PDR versus dm without DR)	VEGF^2^ −936C/T	TT	NA^4^	[45]
Australian population	364 (PDR versus non-PDR versus dm without DR)	VEGF^2^—rs3025021 and rs10434, consecutively	C allele rs3025021 G allele	3.8 (0.002) 2.6 (0.002)	[46]
Japanese population	364 (PDR versus dm without DR)	VEGF^2^ −634C/G; −2578C/A	No association; AA genotype	No association; 7.7 (0.002)	[47]
Han Chinese population	285 (PDR versus non-PDR versus dm without DR)	VEGF^2^ −634C/G	No association	NA^4^ (0.6)	[48]
South Korean population	387 (PDR versus non-PDR versus dm without DR)	rs699947, rs1570360, and rs2010963—VEGF^2^	AGG haplotype	4.3 (*P* = 0.019)	[49]
Iranian population	398 (PDR versus NDR)	VEGF^2^ +405	GG	1.87 (*P* = 0.039)	[50]

^1^Odds ratio and *P* value in logistic regression analysis; ^2^vascular endothelial growth factor; ^3^transforming growth factor-beta; ^4^not available; ^5^cytosine-adenine (CA)_(*n*)_ repeat in the promoter of the insulin-like growth factor (IGF) gene;^ 6^basic fibroblast growth factor.

**Table 3:** The reported studies of polymorphisms of genes of oxidative stress and proliferative diabetic retinopathy in subjects with type 2 diabetes.

Population	Subjects with type 2 diabetes (*n*)	Polymorphism (rs)	Risk genotype/allele	OR (*P* value)^1^	Reference
Caucasian-Brazilians	501 (PDR versus NDR versus dm without DR)	UCP2^2^: −866G/A^3^; Ala55Val^4^; 45 bp Ins/Del^5^	Haplotype A Val Ins	2.12 (0.006)	[53]
Slovene population (Caucasians)	577 (PDR versus dm without PDR)	eNOS 4a/4b; eNOS^6^ 894G>T^3^ eNOS^6^	aa; none	2.9 (=0.005); no association	[19]
Chinese population	8111 (PDR versus NDR versus dm without DR)	eNOS^6^ 4a/4b; eNOS 894G>T^7^ eNOS; T −786C eNOS^8^	No association (4a/b; 894G>T); 786C—antirisk	No association (4a/b; 894G>T); 786C—antirisk	[54]
Caucasian-Brazilians	630 (PDR versus NDR versus dm without DR)	eNOS^2^ 4a/4b; eNOS^2^ 894G>T^3^ eNOS^2^; T −786C eNOS^3^	No association	No association	[55]

^1^Odds ratio and *P* value in logistic regression analysis; ^2^uncoupling protein 2; ^3^866G/A (rs659366); ^4^Ala55Val (rs660339); ^5^insertion/deletion (Ins/Del) polymorphism; ^6^endothelial nitric oxide synthase; ^7^894G>T (Glu298Asp).

**Table 4:** The reported studies of the genes involved in iron metabolism and proliferative diabetic retinopathy in subjects with type 2 diabetes.

Population	Subjects with type 2 diabetes (*n*)	Polymorphism (rs)	Risk genotype/allele	OR (*P* value)^1^	Reference
Slovene population (Caucasians)	223 (PDR versus dm without PDR)	C282Y-HFE^2^; H63D-HFE^2^	C282Y heterozygosity; no association	3.0 (0.02); NA	[57]
USA study^3^	2052 (PDR versus NDR versus dm without DR)	EPO^4^ promoter-rs1617640	T allele	2.0 (<0.001)	[56]
Australian^5^ population	345 (PDR versus NDR versus dm without DR)	EPO^4^-rs507392; rs1617640; rs551238	CC/GG/CC; consecutively	<0.008; <0.008; <0.008; consecutively	[58]

^1^Odds ratio and *P* value in logistic regression analysis; ^2^hemochromatosis gene; ^3^three European-American cohorts (Utah; GoKinD Study; and Boston). ^4^Erythropoietin gene; ^5^ninety-three percent were Caucasians of European descent, 7% were of Asian and Middle Eastern descent.

**Table 5:** The reported studies of polymorphisms of the cytokines genes and proliferative diabetic retinopathy in subjects with type 2 diabetes.

Population	Subjects with type 2 diabetes (*n*)	Polymorphism (rs)	Risk genotype/allele	OR (*P* value)^1^	Reference
Slovak population (Caucasians)	246 (PDR versus dm without PDR)	TNF^2^-*β* NcoI	*β*2 allele	NA (<0.01)	[59]
Asian Indian population	207 (PDR versus dm without DR)	TNF^2^ (GT)_*n*_ microsatellite^3^	Allele 8 (111 bp)	NA (<0.01)	[60]
Japanese population	251 (PDR versus NDR versus dm without DR)	LTA^4^ −804C/A, 252A/G; TNF^2^ *α* (−302A/G)	Genotype distribution	No association	[61]
Indian population	493 (PDR versus dm without DR)	IL^5^-10 1082G allele; TNF^2^ *α* −238A	GG genotype; AA genotype	2.2 (0.0037); 5.8 (0.001)	[62]
Korean population	590 (PDR versus dm without PDR)	MCP-1^6^ −2518A/G	AA genotype	1.9 (0.009)	[63]

^1^Odds ratio and *P* value in logistic regression analysis; ^2^tumor necrosis factor; ^3^(GT)*n* microsatellite dinucleotide repeat upstream to the promoter region of TNF gene; lymphotoxin *α*  −804C/A polymorphism in exon 3 and 252A/G polymorphism in intron 1; ^5^interleukin; ^6^monocyte chemoattractant protein-1.

**Table 6:** The reported studies of adhesion molecules (ICAM, PECAM, VCAM) and proliferative diabetic retinopathy in subjects with type 2 diabetes.

Population	Subjects with type 2 diabetes (*n*)	Polymorphism (rs)	Risk genotype/allele	OR (*P* value)^1^	Reference
Chinese population	172 (PDR versus NDR versus dm without DR)	ICAM-1^2^-K469E ICAM-1^2^-G241A	K allele; no association	NA (0.01); NA	[64]
Slovene population (Caucasians)	338 (PDR versus dm without DR)	ICAM-1^2^-K469E ICAM-1^2^-G241A	EE; no association	2.0 (0.01); NA	[18]

^1^Odds ratio and *P* value in logistic regression analysis; ^2^intracellular adhesion molecule-1.

**Table 7:** The reported studies of polyol pathway (aldose reductase/sorbitol dehydrogenase) genes and proliferative diabetic retinopathy in subjects with type 2 diabetes.

Population	Subjects with type 2 diabetes (*n*)	Polymorphism (rs)	Risk genotype/allele	OR (*P* value)^1^	Reference
Japanese population	61 (PDR versus nonPDR)	AR^2^ (AC)_*n*_	z-4		[65]
Brazilian population	579 (PDR versus non-PDR)	−106C>T AR^2^	CC	2.04 (*P* = 0.007)	[66]
Poland (Caucasians)	215 (PDR versus non-PDR versus dm without DR)	−SDH^3^ C −1214G (rs2055858) and G −888C (rs3759890)	None	—	[67]

^1^Odds ratio and *P* value in logistic regression analysis; ^2^aldose reductase; ^3^sorbitol dehydrogenase.

**Table 8:** The reported studies of the renin-angiotensin system and proliferative diabetic retinopathy in subjects with type 2 diabetes.

Population	Subjects with type 2 diabetes (*n*)	Polymorphism (rs)	Risk genotype/allele	OR (*P* value)^1^	Reference
Danish population (Caucasians)	222 (PDR versus dm without DR)	ACE I/D^2^	DD—no association	1.2 (0.4)	[68]
Slovene population (Caucasians)	204 (PDR versus NDR versus dm without DR)	ACE I/D^2^	DD—no association	1.2 (0.4)	[69]
Chinese population	4385 (PDR versus NDR versus dm without DR)	ACE I/D^2^	DD—no association	NA	[70]
Chinese population	2224 (PDR versus dm without DR)	ACE I/D^2^	DD	2.2 (<0.01)	[71]

^1^Odds ratio and *P* value in logistic regression analysis; ^2^insertion/deletion polymorphism of the angiotensin I-converting enzyme gene.
